# A fast-responsive two-photon fluorescent probe for monitoring endogenous HClO with a large turn-on signal and its application in zebrafish imaging†

**DOI:** 10.1039/c9ra02160d

**Published:** 2019-05-28

**Authors:** Jian-Yong Wang, Jianbo Qu, Haitao Zhang, Kang Wei, Shan-Xiu Ni

**Affiliations:** School of Light Industry and Engineering, Qilu University of Technology (Shandong Academy of Sciences) Jinan 250353 P. R. China wjy@qlu.edu.cn

## Abstract

A novel fast-responsive two-photon fluorescent probe NS-ClO was constructed for imaging endogenous HClO in living cells, tissues and fresh zebrafish with a large turn-on signal (about 860 times) and Stokes shift (about 90 nm). The probe NS-ClO for the recognition of HClO *in vivo* exhibited fast response (about 1 min) and good selectivity; thus, it might be a useful tool to understand the role of HClO in various physiological processes.

## Abstract 2

Fast-responsive two-photon fluorescent probe NS-ClO for imaging endogenous HClO *in vivo* with a large turn-on signal (about 860 times) and Stokes shift (about 90 nm), fast response (about 1 min) and good selectivity.

## Graphical abstract

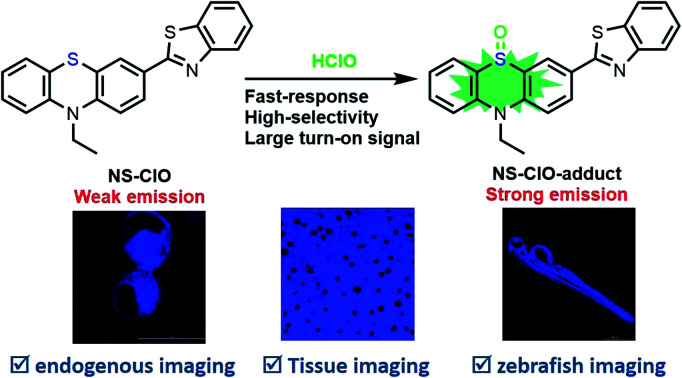

Hypochlorous acid (HClO) is a weak acid with oxidizing properties in the reactive oxygen species (ROS) family and it is generated from living immunological cells by oxidising hydrogen peroxide (H_2_O_2_) and chloride with the help of myeloperoxidase (MPO).^1^ HClO has an important influence on various physiological processes including immune defence against microorganisms and the lethal effect on pathogens in living biosamples. When the balance of HClO in the body is destroyed, many molecules such as DNA, RNA, fatty acids, cholesterol, and proteins can react with HClO, which is related to different diseases including neurodegenerative disorders and cancers.^2–5^ Further research between the HClO level and the pathophysiological process is very necessary. Therefore, it is important to develop a practicable method for monitoring HClO in a physiological atmosphere.

In the past few decades, many efficient and functional methods including colorimetric methods, chemiluminescence methods, coulometry, radiolysis, and electrochemical and chromatographic methods were applied to monitor HClO.^6–11^ Although the mentioned methods exhibit fast responses and are selective to HClO over other molecules, sophisticated equipment and complex operating techniques are needed in the processes. Also, the living biosystem can often be damaged in the operation. Hence, they are not suitable for detecting HClO in living cells, tissues, and body. During the last few years, an organic molecular probe has been the most useful detection tool and it is efficient for the real-time visualization of small bioactive molecules in the living biosystem with high selectivity and sensitivity; this facilitates comprehensive exploration and manipulation in the physiological atmosphere.^12–21^

Recently, many fluorescent probes, acting as an inevitable tool for monitoring HClO in the living biosystem, have been constructed.^22–26^ Although most of the previous probes were used to image exogenous HClO, it is still difficult to perform endogenous imaging of HClO in living cells. Especially, an organic fluorescent probe with a large turn-on Stokes shift and intensity, two-photon excitation, fast response, and good selectivity and stability is still scarce. Therefore, it is worth to develop a two-photon fluorescent probe for imaging HClO specifically *in vivo* with a large Stokes shift and a turn-on signal.

In this work, the modified organic fluorescent probe NS-ClO was constructed for imaging endogenous HClO specifically with a large turn-on signal (about 860 times) and Stokes shift (about 90 nm) (Scheme 1). This turn-on fluorescent probe NS-ClO with good properties including two-photon excitation, fast response (about 1 min) and good selectivity was studied; this method might be useful to monitor the functions of HClO in various physiological processes (Table S1†).

**Scheme 1:**
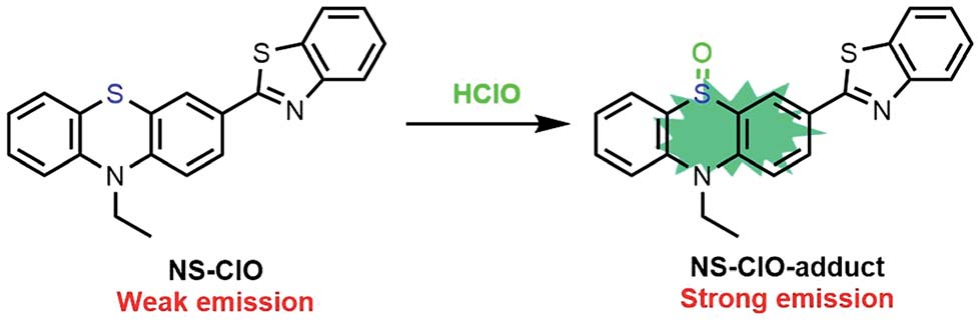
The structure of NS-ClO and the proposed sensing mechanism for HClO.

The sulfur atom in phenothiazine is very reactive towards HClO. The fluorescence property of phenothiazine was changed by oxidising a sulfur atom to sulfoxide, as depicted in Scheme 1. Benzothiazole is a very common electron-withdrawing group and is stable under oxidation and reduction conditions, which ensured that the constructed probe is stable in excessive HClO. Herein, the probe NS-ClO was developed by introducing benzothiazole into phenothiazine with the sulfur atom as a recognition site to HClO in one step easily. The characterization of the probe NS-ClO by ^1^H NMR, ^13^C NMR and HRMS was performed, and the details are provided in the ESI.†

The spectral properties of probe NS-ClO were studied. There was almost no fluorescence intensity of probe NS-ClO at 450 nm in PBS buffer (pH = 7.4) and DMF (v/v = 19/1) at an ambient temperature without the addition of HClO (Fig. 1). When different concentrations of HClO were added to the reaction system, a maximal absorption band appeared at around 360 nm and strong fluorescence emission was observed, as shown in Fig. S1† and 1; also, a large Stokes shift (about 90 nm) was seen. Therefore, PBS buffer (pH = 7.4) containing DMF (v/v = 19/1) was considered as the best solvent for this experiment. In addition, we also found that the two-photon probe NS-ClO was stable in the presence of excess HClO (20 equiv.) when the time was extended to 8 min (Fig. 1c and d).

**Fig. 1:**
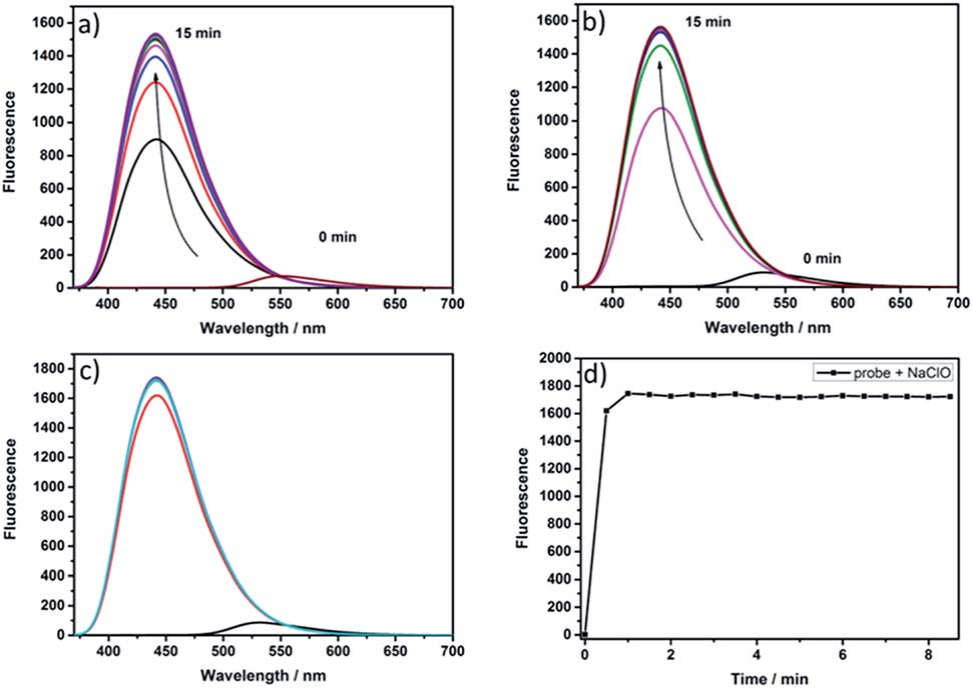
Reaction-time profiles of NS-ClO (5 μM) in the absence or presence of NaClO: (a) NaClO (8.0 equiv.); (b) NaClO (10.0 equiv.); (c) NaClO (20.0 equiv.); (d) NaClO (20.0 equiv.).

When the probe NS-ClO was excited at 360 nm, there was almost no fluorescence (*φ* = 0.03) using a fluorescein (*φ*_r_ = 0.90 in 0.1 N NaOH) solution.^27^ However, when different amounts of HClO were added, the obvious turn-on fluorescence enhancement (about 860-fold) exhibited a quantum yield of 0.57 at 450 nm with a detection limit of 0.75 μM (Fig. S2†). Therefore, this two-photon probe NS-ClO exhibited a fast response and large turn-on enhancement. In addition, the possible sensing mechanism was studied by mass spectrometry. When the probe NS-ClO (20 μM) was dissolved in PBS buffer (pH = 7.4) and DMF (v/v = 19/1), excess of HClO was added to the previous solvent. The spectra show a clear peak at *m*/*z* 377.0774, corresponding to the NS-ClO-adduct (Fig. S4†); this was in good agreement with the possible sensing mechanism reported in a previous work^28^ (Scheme 1).

Another important factor, *i.e.*, the pH of PBS buffer was examined, which may have significant impact on the response to HClO when PBS buffers having different pH values were used to examine the fluorescence intensity of this probe. There were almost no changes in the absence of HClO when the pH value changed from acidic to basic (about 1.0 to 10.0). With the addition of HClO (5.0 equiv.), the fluorescence intensity gradually increased when the pH was changed from 1.0 to 8.5 and rapidly decreased from 8.5 to 10.0. The main reason is that the oxidizing properties of HClO decline significantly in alkaline conditions. However, we found that the probe NS-ClO could detect HClO in PBS buffer (pH = 7.4) containing DMF (v/v = 19/1) at the physiological pH (7.4) with about 860-fold enhancement. In other words, this probe can be suitable for biological applications (Fig. 2 and 3).

**Fig. 2:**
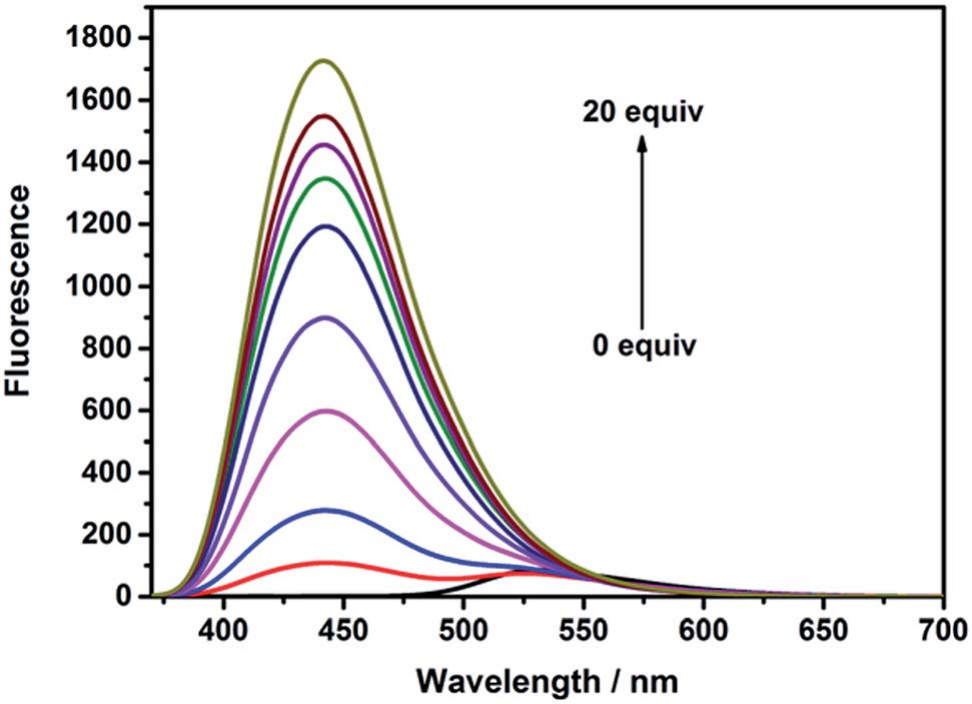
The fluorescence spectra of NS-ClO (5 μM) in pH 7.4 PBS buffer (containing 5% DMF) in the absence or presence of NaClO (0–20 equiv.).

**Fig. 3:**
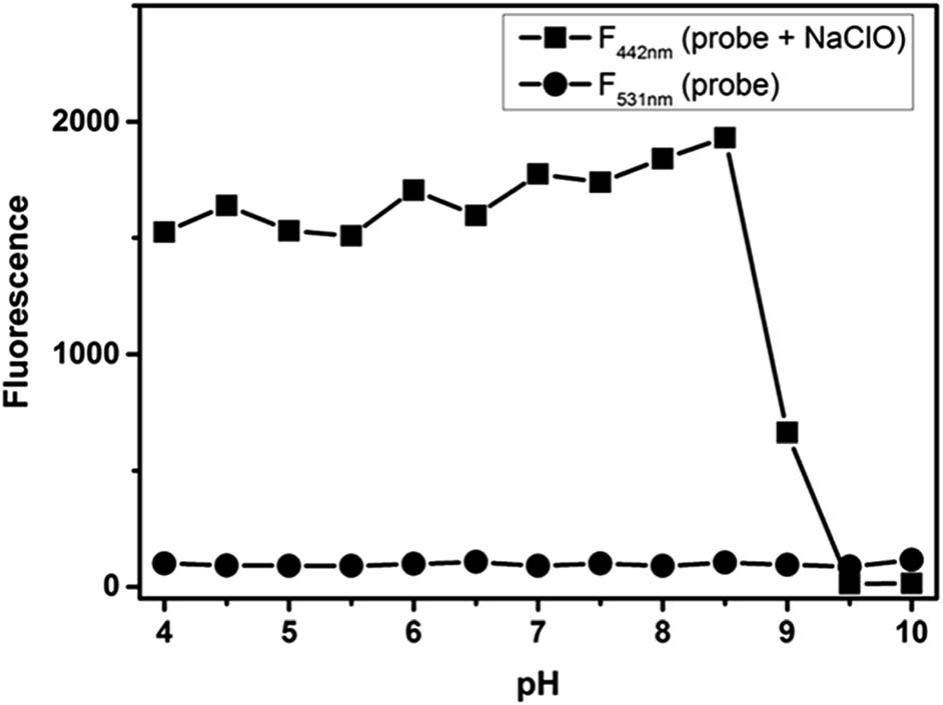
The pH effects of fluorescence spectra of NS-ClO (5 μM) in pH 7.4 PBS buffer (containing 5% DMF) in the absence (●) or presence (■) of NaClO (5.0 equiv.).

In order to investigate selectivity, the two-photon probe NS-ClO was reacted with distinct biologically reactive analytes including biological thiols, reactive oxygen species (ROSs), reactive nitrogen species (RNSs) and anions. As listed in Fig. 4, the fluorescence enhancement is basically unchanged with the addition of different species (GSH, Cys, Hcy, F^−^, Cl^−^, Br^−^, ·OH, ONOO^−^, DTBP, TBHP, NO, H_2_O_2_, NO_2_^−^, Co^+^, Cu^2+^, and Ni^+^). However, when we added HClO to the detection system, the fluorescence enhancement increased significantly within 1 min. This result indicated that the NS-ClO probe can be applied to monitor HClO with good selectivity compared to other different species (Scheme 2).

**Fig. 4:**
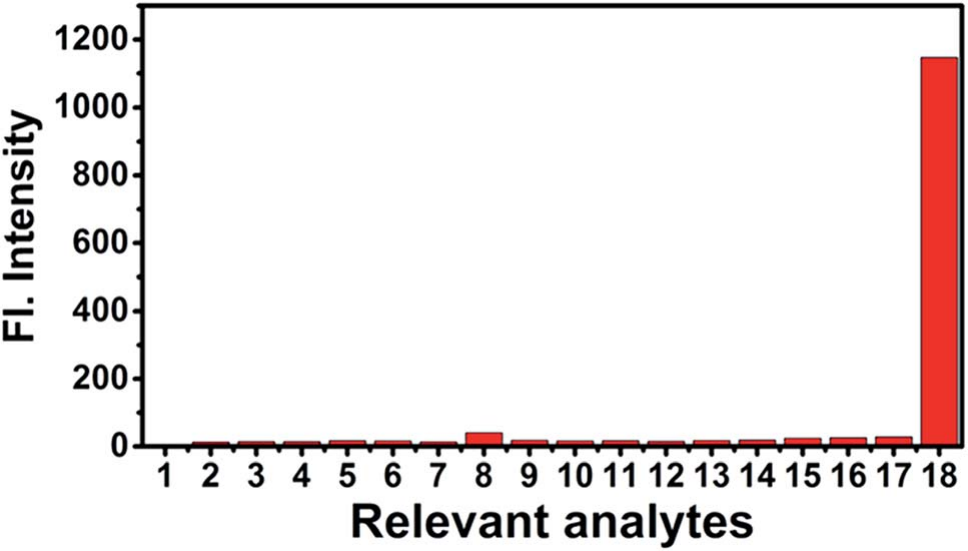
Fluorescence spectra of NS-ClO (10 μM) in pH 7.4 PBS buffer (containing 5% DMF) for various relevant species (50 μM). 1: None; 2: GSH; 3: Cys; 4: Hcy; 5: F^−^; 6: Cl^−^; 7: Br^−^; 8: ·OH; 9: ONOO^−^; 10: DTBP; 11: TBHP; 12: NO; 13: H_2_O_2_; 14: NO_2_^−^; 15: Co^2+^; 16: Cu^2+^; 17: Ni^2+^; 18: ClO^−^.

**Scheme 2:**
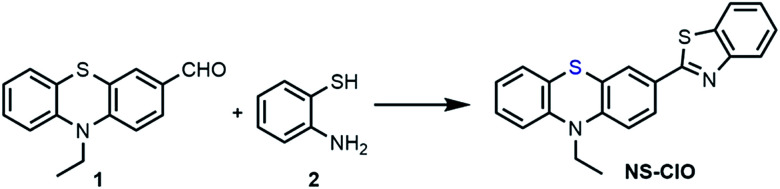
Synthesis of the two-photon fluorescent probe NS-ClO.

Encouraged by the above-mentioned excellent results, we inferred that the highly sensitive and selective two-photon probe NS-ClO could be suitable for imaging HClO in the living biosystem. First, the results of MTT assays proved that the HeLa cell survival rate is very high after treatment with different concentrations of NS-ClO. That is to say, the probe NS-ClO exhibited low cytotoxicity to HeLa cells after one day even at high concentrations (30.0 μM) (Fig. S5†) and could be applied to monitor HClO in living cells. We investigated the applicability of probe NS-ClO for monitoring exogenous HClO in HeLa cells. As depicted in Fig. 5, living HeLa cells are initially treated with probe NS-ClO (10 μM) for 30 min and washed three times with PBS buffer for removing excess probe NS-ClO. The experimental data indicated that the HeLa cells incubated with probe NS-ClO exhibited almost no fluorescence in the blue channel (Fig. 5b). However, when the living HeLa cells were treated with probe NS-ClO for 30 min and NaClO (30 μM) for another 30 min, the fluorescence signal emerged obviously in the blue channel (Fig. 5e). Therefore, the probe NS-ClO with good membrane permeability can be used for imaging exogenous HClO in living HeLa cells.

**Fig. 5:**
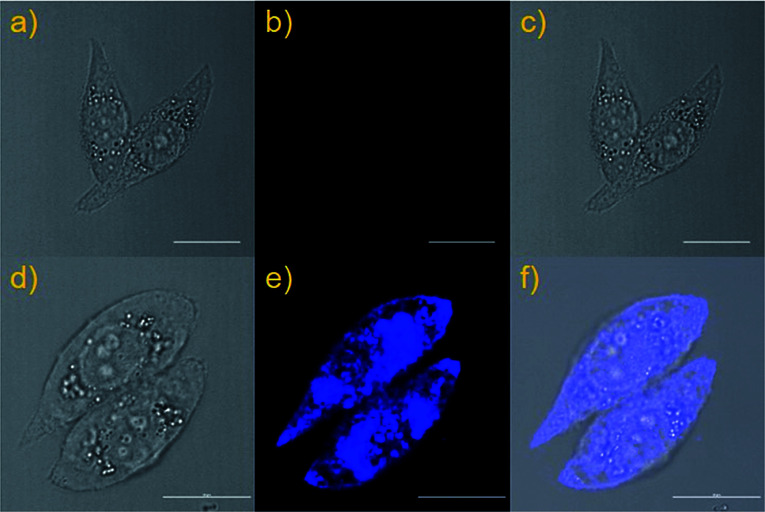
Imaging of exogenous HClO in HeLa cells stained with the probe NS-ClO (10 μM). (a) Bright-field image of HeLa cells co-stained only with NS-ClO; (b) fluorescence images of (a) from blue channel; (c) overlay of the bright-field image (a) and blue channel (b). (d) Bright-field image of HeLa cells co-stained with NS-ClO and treated with NaClO. (e) Fluorescence images of (d) from blue channel; (f) overlay of the bright-field image (d) and blue channel (e).

The above-mentioned data proved that the developed probe NS-ClO can be used for exogenous imaging. Therefore, the endogenous detection of HClO was completed subsequently in murine live macrophage cell line RAW 264.7. According to previous reports,^29^ lipopolysaccharide (LPS) and phorbol myristate acetate (PMA) are added to stimulate macrophages to produce endogenous HClO. As depicted in Fig. 6, the living RAW 264.7 macrophage cells pre-loaded with probe NS-ClO (10 μM) show almost no fluorescence signal in the blue channel (Fig. 6b). However, when the living RAW 264.7 cells were exposed to LPS (2 μg mL^−1^) and PMA (2 μg mL^−1^) together and then treated with probe NS-ClO (10 μM), obvious fluorescence enhancement was obtained (Fig. 6e). The result demonstrated that the constructed two-photon probe NS-ClO is suitable for monitoring endogenous HClO in living RAW 264.7 macrophage cells.

**Fig. 6:**
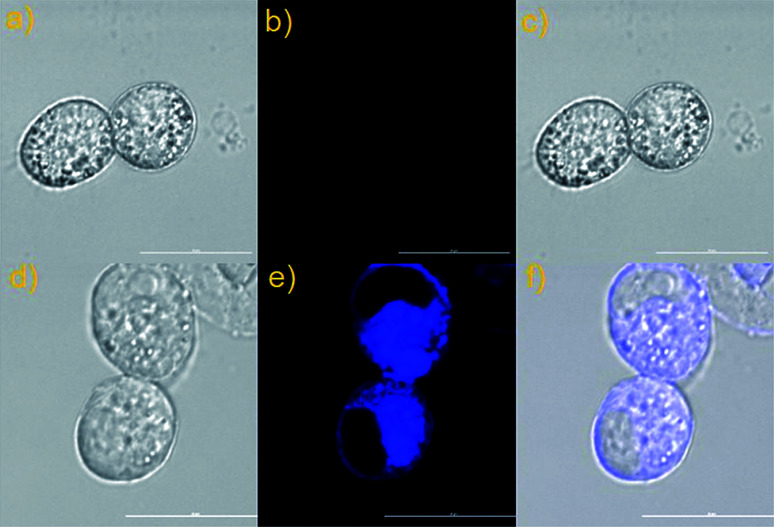
Imaging of endogenous HClO in RAW 264.7 cells stained with the probe NS-ClO. (a) Bright-field image of RAW 264.7 macrophage cells co-stained with NS-ClO. (b) Fluorescence images of (a) from blue channel; (c) overlay of (a) and (b). (d) Bright-field image of stimulated RAW 264.7 macrophage cells co-stained with NS-ClO, PMA and LPS. (e) Fluorescence images of (d) from blue channel; (f) overlay of the bright-field image (d) and blue channels (e).

Previous research indicates that the probe NS-ClO can be used for imaging HClO *in vitro* and *in vivo*. With these data and advantages of TPM in hand, two-photon fluorescence imaging of HClO in living mouse tissues by TPM was performed with probe NS-ClO. Living tissue slices of mouse liver of about 400 μm thickness were prepared. Initially, the prepared tissues were washed with PBS buffer and treated with the probe NS-ClO (10.0 μM) for 30 min at 37 °C. After scanning by TPM, there was no fluorescence signal in the blue channel (Fig. 7a). On the contrary, when fresh tissues were pre-treated with the probe NS-ClO (10.0 μM) for 30 min and incubated with NaClO (10.0 μM) for another 30 min, an obvious fluorescence signal was observed from 5 to 80 μm depth (Fig. 7b). These excellent merits suggest that the turn-on probe NS-ClO can be used for tissue imaging with two-photon excitation.

**Fig. 7:**
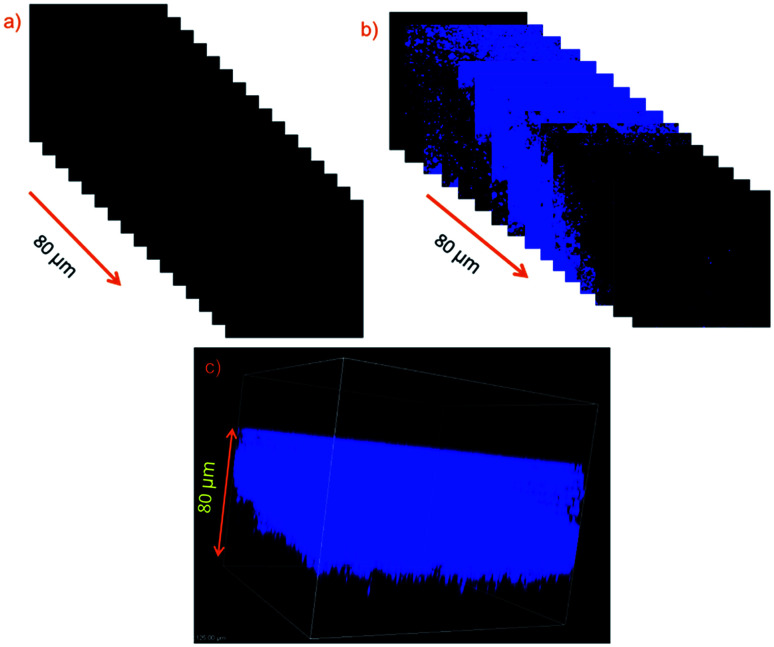
(a) Two-photon fluorescence images of a fresh mouse liver slice incubated with NS-ClO probe (10.0 μM) for 30 min in PBS buffer exhibiting no fluorescence at the emission window of 0–80 nm. (b) Two-photon fluorescence images of a fresh mouse liver slice pretreated with NS-ClO (10 μM) and NaClO (10 equiv.) in PBS buffer at the depths of approximately 0–80 μm. (c) The three-dimensional image of (b). Excitation at 800 nm with fs pulse.

Because the transparent nature of zebrafish appears in all stages of embryonic growth, the imaging of zebrafish was considered to be a very physiological vertebrate model for the detection of HClO.^30^ Due to the advantages of two-photon excitation, further investigation of probe NS-ClO to monitor HClO in living zebrafish was carried out. When a 5 day-old zebrafish was treated with probe NS-ClO, no fluorescence appeared in the blue channel (Fig. 8b). However, after further treatment with NaClO for 20 min, the fluorescence signal at around two zygomorphic areas around the yolk extension and eyes of the living zebrafish obviously emerged, as shown in Fig. 8e. The result indicated that the developed probe NS-ClO can be used for zebrafish imaging.

**Fig. 8:**
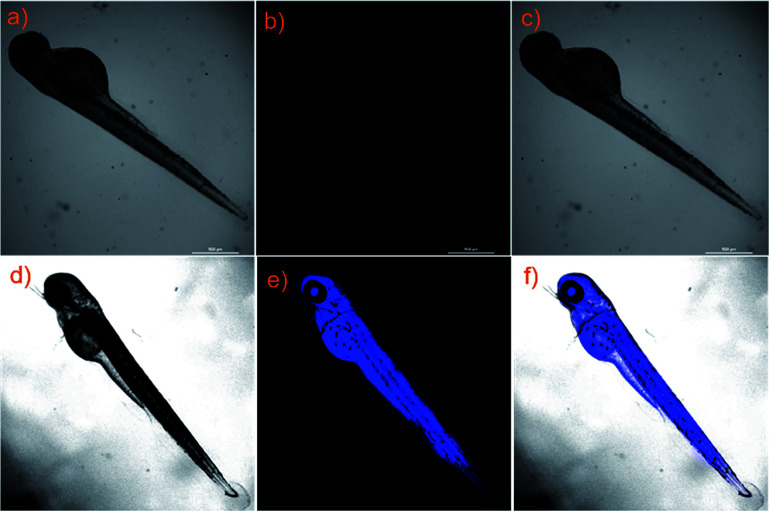
Imaging of HClO in zebrafish stained with the probe NS-ClO (a) Bright-field image of zebrafish costained with NS-ClO; (b) fluorescence images of (a) from blue channel; (c) overlay of (a) and (b). (d) Bright-field image of zebrafish costained with NS-ClO and treated with NaClO. (e) Fluorescence images of (d) from blue channel; (f) overlay of the bright-field image (d) and blue channels (e).

In conclusion, a fast-responsive fluorescent probe with two-photon excitation, a large turn-on signal (about 860 times) and a large Stokes shift (about 90 nm) for the detection of HClO *in vivo* was developed. The ideal probe NS-ClO exhibited good properties including excellent selectivity, high sensitivity (about 1 min) and low cytotoxicity. In addition, the two-photon probe NS-ClO could be used for the detection of HClO in living cells, tissues and fresh zebrafish *in vivo*. Therefore, the probe NS-ClO can be developed into other functional two-photon probes for the recognition of other analytes and can be applied to investigate the biological and pathological functions of HClO in living biosamples.

## Ethical statement

All animal procedures were performed in accordance with the Guidelines for the Care and Use of Laboratory Animals of Shandong University and experiments were approved by the Animal Ethics Committee of Shandong University according to the requirements of the National Act on the use of experimental animals (China).

## Conflicts of interest

There are no conflicts to declare.

## Supplementary Material

RA-009-C9RA02160D-s001
