# Supplementary material for: A fast-responsive two-photon fluorescent probe for monitoring endogenous HClO with a large turn-on signal and its application in zebrafish imaging
Source: RSC Adv. 2019 May 28;9(29):16467–71. doi: 10.1039/c9ra02160d (PMC9064391; doi:10.1039/c9ra02160d)
Supplement: RA-009-C9RA02160D-s001 [file RA-009-C9RA02160D-s001.pdf]

Electronic Supplementary Material (ESI) for RSC Advances.
This journal is © The Royal Society of Chemistry 2019

Supporting Information for

A fast-responsive two-photon fluorescent probe for monitoring endogenous HClO with a large turn-on signal and its application in zebrafish imaging

Jian-Yong Wang, Jianbo Qu, Haitao Zhang, Kang Wei, Shan-Xiu Ni*

School of Light Industry and Engineering, Qi Lu University of Technology (Shandong Academy of Sciences), Jinan, 250353, P. R., China,

Email: wjy@qlu.edu.cn

*Correspondence to: Jian-Yong Wang, School of Light Industry and Engineering, Qi Lu University of Technology(Shandong Academy of Sciences), Jinan, 250353, P. R., China, Email: wjy@qlu.edu.cn

Table of contents

Materials and instruments………… ……………………………………………….S3

Determination of the fluorescence quantum yield. ………………………………S3

Cell culture…. . . ………………………………………………………….……….S4

Cytotoxicity assays ……………………………………………………….……….S4

Imaging of exogenous HClO in HeLa cells ……………………………………..S4

Imaging of endogenous HOCl in RAW 264.7 cells …………………………..S5

Preparation of fresh mouse liver slices and two-photon fluorescence imaging….S5

Imaging of HClO in zebrafish ……………………………………………………S5

Synthesis of the probe NS-ClO ……………………………………….………. S6

Fig. S1………………………… ………………………………………….….….S7

Fig. S2….………………………… ……………………………………………….S7

Fig. S3….…………………………………………………………………….……….S7

Fig. S4….…………………………………………………………………….……….S8

Fig. S5….……………………………………………………………………………..S8

Fig. S6….……………………………………………………………………………..S9

Table S1……………………………………………………………………………..S9

Materials and instruments

Without other noted, all the solvents, reagents and materials were obtained from business company and used without other purification. Twice-distilled water was applied to all measurements and experiments. High-resolution electrospray mass spectra (HRMS) were gained from Bruker APEX IV-FTMS 7.0T mass spectrometer; NMR spectra were examined from AVANCE III 400 MHz Digital NMR Spectrometer with TMS as an internal standard; Electronic absorption spectra were recorded on a LabTech UV Power spectrometer; Fluorescence spectra were obtained with a HITACHI F4600 fluorescence spectrophotometer; The fluorescence images of cells and tissues were collected with Nikon A1MP confocal microscopy with a CCD camera; The pH measurements were implemented on a Mettler-Toledo Delta 320 pH meter; analysis was exhibited on silica gel plates and column chromatography was carried out over silica gel (mesh 200-300). Both TLC and silica gel were purchased from the Qingdao Ocean Chemicals.

Determination of the fluorescence quantum yield

Fluorescence quantum yields were determined by using fluorescein (0.1 M in NaOH) according to previous report.[1] The fluorescence quantum yield of compound **NS-ClO** and **NS-ClO-adduct** was calculated according to the following equation :

$$\eta_s = \frac{A_r I_s n_s^2}{A_s I_r n_r^2}\eta_r (A \le 0.05)$$

In the equation, s and r represent the sample and the reference (fluorescein) molecule respectively, η represents the fluorescence quantum yield, A is the absorbance of molecules that were controlled below 0.05 at the excitation wavelength for both molecules in the experiment, I means the integrated emission area and n is the refractive index of the solvent.

Cell culture

The living HeLa cells, and RAW 264.7 cells were cultured in the Dulbecco's modified Eagle's medium (DMEM) supplemented with fetal bovine serum (10% FBS) under the atmosphere containing 5% CO_2 and 95% air at 37 °C.

Cytotoxicity assays:

The living cells line were treated in DMEM (Dulbecco's Modified Eagle Medium) supplied with fetal bovine serum (10%, FBS), penicillin (100 U/mL) and streptomycin (100 μg/mL) under the atmosphere of CO_2 (5%) and air (95%) at 37 °C. The HeLa cells were then seeded into 96-well plates, and 0, 1, 5, 10, 20, 30 μM (final concentration) of the probe **NS-ClO** (99.9% DMEM and 0.1% DMSO) were added respectively. Subsequently, the cells were cultured at 37 °C in an atmosphere of CO_2 (5%) and air (95%) for 24 hours. Then the HeLa cells were washed with PBS buffer, and DMEM medium (500 μL) was added. Next, MTT (50 μL, 5 mg/mL) was injected to every well and incubated for 4 h. Violet formazan was treated with sodium dodecyl sulfate solution (500 μL) in the H_2O-DMF mixture. Absorbance of the solution was measured at 570 nm by the way of a microplate reader. The cell viability was determined by assuming 100% cell viability for cells without **NS-ClO**.

Imaging of exogenous HClO in HeLa cells

Before using, the HeLa cells were washed with PBS three times and then incubated with **NS-ClO** (10 μM) for 30 min at an atmosphere of CO_2 (5%) and air (95%) in the condition of 37 °C. After incubating with NaClO (10 μM) for another 0.5 h under the same conditions. Subsequently, the HeLa cells were rinsed by PBS buffers three times. The ideal fluorescence images were obtained by means of Nikon A1MP confocal microscopy with the equipment of a cooled CCD camera.

Imaging of endogenous HOCl in RAW 264.7 cells

The RAW 264.7 cells were plated on 6-well plates and allowed to adhere for one day. And then the cells were incubated with probe **NS-ClO** (10 μM) for 0.5 h at 37 ˚C. After washing with PBS buffer for three times, 2 □g/ml PMA (phorbol 12-myristate13-acetate) and 2 □g/ml LPS (lipopolysaccharides) were added and incubated for another 2 h. As to the control experiments, the RAW 264.7 cells were not treated with PMA/LPS and only incubated with probe **NS-ClO** (10 μM) for 2 h at an atmosphere of CO_2 (5%) and air (95%) in the condition of 37 ˚C. Before the fluorescence imaging, the cells were washed with PBS buffer. The Nikon A1MP confocal microscopy inverted fluorescence microscopy equipped with a cooled CCD camera were used for fluorescence images.

Preparation of fresh mouse liver slices and two-photon fluorescence imaging

The fresh mouse liver slices were obtained from the liver of 14-day-old mouse. The living liver slices were gained with 400 micron thickness using a vibrating-blade microtome in 25 mM PBS (pH 7.4). The fresh liver slices were pre-treated with **NS-ClO** (10 μM) for 0.5 h. Following another incubation with NaClO (100 μM) for 0.5 h in the condition of 37 °C, the slices were washed three times by PBS buffer and imaged. As to the control experiments, the fresh tissues were not treated with NaClO and only incubated with probe **NS-ClO** (10 μM) for 0.5 h at the same conditions. The two-photon fluorescent images were collected with excitation wavelength at 800 nm and emission wavelength at 450 nm by means of Nikon A1MP confocal microscopy inverted fluorescence microscopy equipped with a cooled CCD camera and a femtosecond laser.

Imaging of HClO in zebrafish

The 5-day-old zebrafish was pre-treated with probe **NS-ClO** (10 μM) in embryo media for 0.5 h at 37 °C and then rinsed with PBS buffers for three times. After another incubation with NaClO (100 μM) for 0.5 h, the fresh zebrafish was washed with PBS buffers three times. As to the control group, the zebrafish incubated with

probe **NS-ClO** (10 μM) for 0.5 h at in the same condition. The zebrafish imaging was completed by Nikon A1MP confocal microscopy inverted fluorescence microscopy equipped with a cooled CCD camera.

Synthesis of the probe NS-ClO

Compound **1** (2.0 mmol, 510.0 mg, 1.0 equiv) and **2** (2.0 mmol, 340.0 mg, 1.0 equiv) were dissolved in DMF (15.0 mL). And the $Na_2S_2O_5$ (2.0 mmol, 380.0 mg, 1.0 equiv) was added under N_2. The reaction was refluxed for 5 h. Then, the distilled water was added (10.0 mL) for extracting with DCM. The product was purified by silica column chromatography to give the two-photon fluorescent probe **NS-ClO** with 60% yield. ^1H NMR (400 MHz, $CDCl_3$) δ 8.05 (d, *J* = 8.1 Hz, 1H), 7.92 - 7.84 (m, 1H), 7.82 (d, *J* = 2.1 Hz, 1H), 7.51 - 7.44 (m, 1H), 7.40 - 7.33 (m, 1H), 7.19 - 7.10 (m, 1H), 6.97 - 6.86 (m, 1H), 3.97 (q, *J* = 7.0 Hz, 1H), 1.45 (t, *J* = 7.0 Hz, 1H). ^{13}C NMR (101 MHz, DMSO-d_6) δ 171.4, 158.8, 152.1, 148.4, 139.4, 133.2, 132.4, 132.3, 132.2, 131.8, 130.4, 130.2, 128.7, 128.3, 127.7, 127.5, 127.0, 121.0, 120.8, 46.7, 17.7; HRMS (ESI) m/z calcd for $C_{19}H_{16}NO_2^+$ $(M+H)^+$: 361.0876; found 361.0859.

Reference

1. J. N. Demas, A. Crosby, *J. Phys. Chem.*, 1971, **75**, 991-1024;

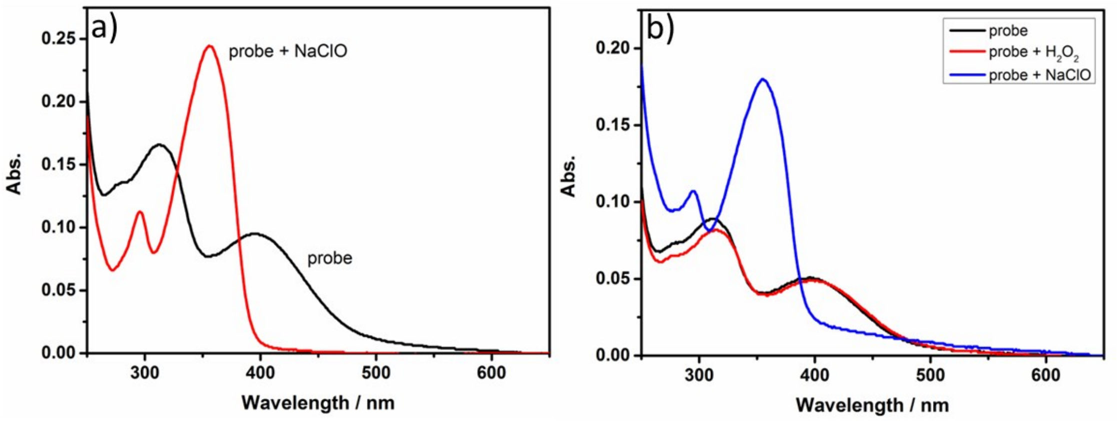

Fig. S1. The absorption spectra of **NS-ClO** (5 μM) in pH 7.4 PBS/DMF (v/v = 95/5) in the absence or presence of H_2O_2 (5 equiv) and NaClO (5 equiv).

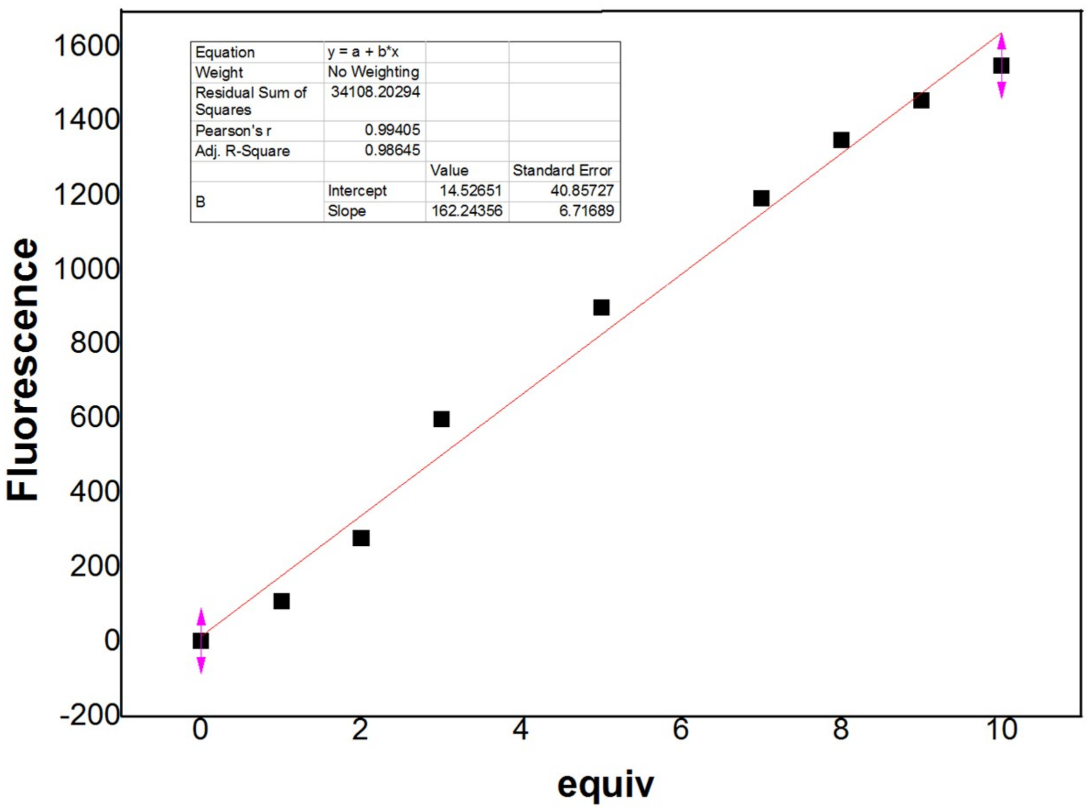

Fig. S2. The linear fit of **NS-ClO** (10 μM) in pH 7.4 PBS buffer (5% DMF) in the absence or presence of NaClO (0-10 equiv).

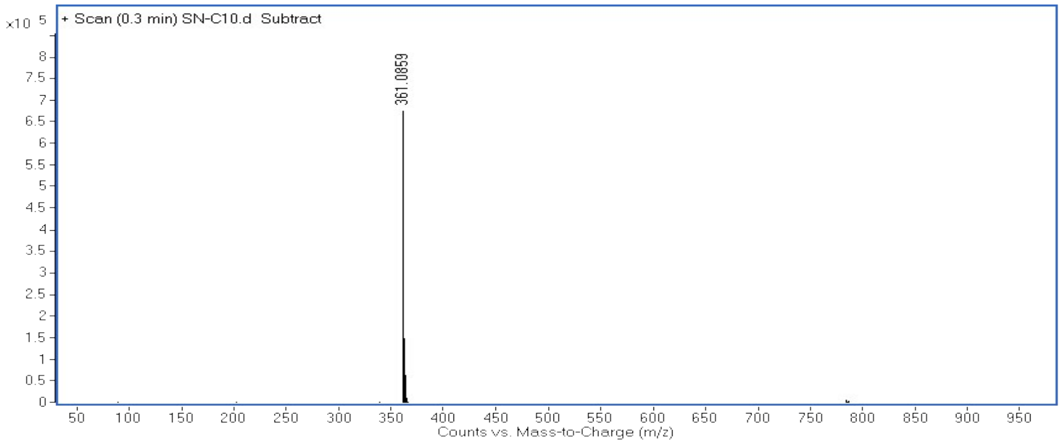

Fig. S3. HRMS (positive ion mode) spectrum of **NS-ClO**.

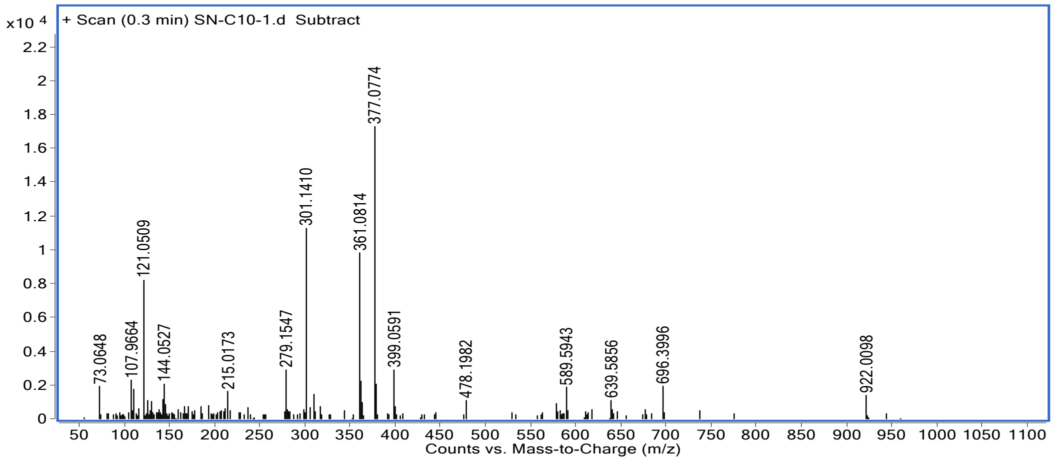

Fig. S4. HRMS (positive ion mode) spectrum of **NS-ClO** (20 µM) after treatment with NaClO (200 µM) in pH 7.4 PBS/DMF (1: 1) for 20 min. The peak at m/z 377.0774 corresponds to **NS-ClO**-adduct.

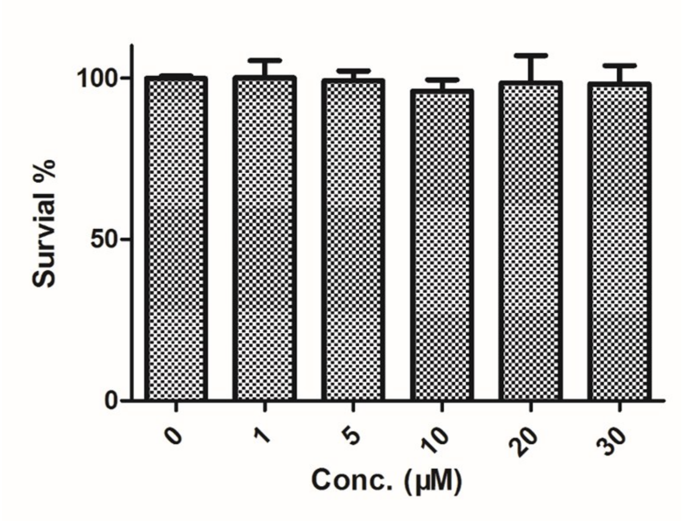

Fig. S5. Cytotoxicity assays of **NS-ClO** at different concentrations (0 µM; 1µM; 5 µM; 10 µM; 20 µM; 30 µM) for HeLa cells

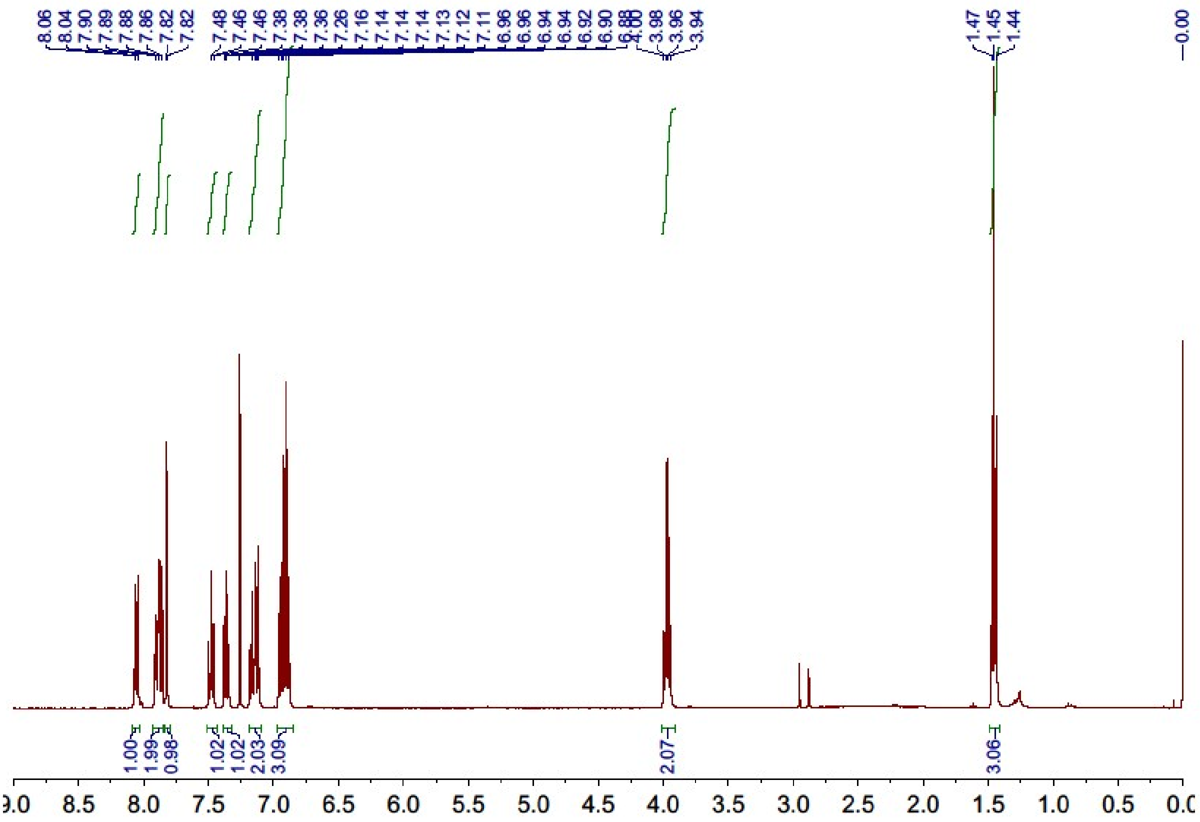

Fig. S5. ^{1}H NMR (DMSO-d_6) spectrum of **NS-ClO.**

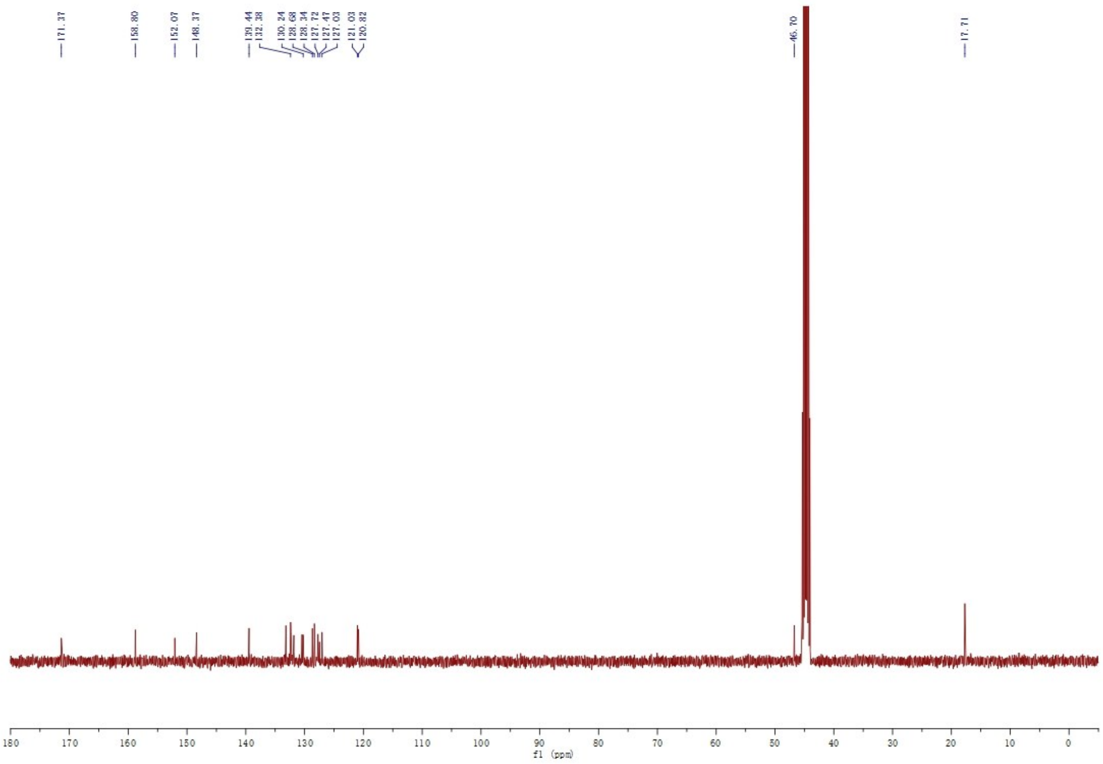

Fig. S6. ^{13}C-NMR (DMSO-d_6) spectrum of **NS-ClO**.

Table S1 Comparison of the reported fluorescent probes based on phenothiazine for the detection of HClO

Probes	Response time	Detection limition	Stokes shift	One or two photon	Imaging	References
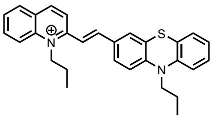	Few seconds	15.6 nM	128 nm	One photon	Living cells Zebrafish mice	Chem. - Asian J., 2018, 13, 2611-2618
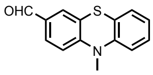	10 s	449.76 nM	168nm	One photon	Living cells	Dyes Pigments, 2019, 162, 160-167.
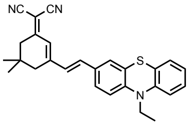	10 s	39 nM	170nm	One photon	Endogenous Living cells	New J. Chem., 2018, 42, 5135-5141
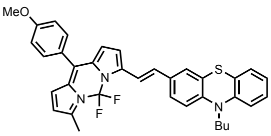	--	4.1 nM	none	One photon	Endogenous Living cells	Sensor. Actuat. B: Chem., 2018, 263, 137-142
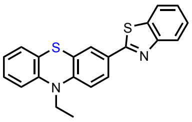	1 min	0.76 μM	90 nm	Two photon	Endogenous Living cells Tissues zebrafish	This work
